# Cannabinoid-Induced Brugada Syndrome: A Case Report

**DOI:** 10.7759/cureus.8615

**Published:** 2020-06-14

**Authors:** Scott C Stockholm, Adam Rosenblum, Alex Byrd, Esteban Mery-Fernandez, Manoj Bhandari

**Affiliations:** 1 Internal Medicine, Cape Fear Valley Hospital, Fayetteville, USA; 2 Internal Medicine, Campbell University School of Osteopathic Medicine, Fayetteville, USA; 3 Internal Medicine, Cape Fear Valley Health System, Fayetteville, USA; 4 Critical Care Medicine, Cape Fear Valley Health System, Fayetteville, USA; 5 Cardiology, Cape Fear Valley Health System, Fayetteville, USA

**Keywords:** brugada, marijuana, pacemaker, cardiac death, ventricular fibrillation, torsade de pointes, substance abuse, autosomal dominant, electrocardiogram (ecg/ekg), young adult male

## Abstract

Brugada syndrome, also called Pokkuri Death Syndrome, is an autosomal dominant electrophysiological phenomenon that increases the risk of spontaneous ventricular tachyarrhythmia and sudden cardiac death. Due to sodium channel mutations in the cardiac membrane, most commonly SCN5A and SCN10A, the heart can be triggered into a fatal arrhythmia. Brugada syndrome can be triggered by fever, and medications including antiarrhythmics, psychotropics, and recreational drugs like cocaine and marijuana. We report a case that demonstrates the diagnosis of Brugada syndrome in an otherwise very healthy 22-year-old African-American male. He presented after a syncopal event and developed spontaneous ventricular tachycardia and torsades de pointes. Electrocardiogram (EKG) findings documented a type I Brugada pattern and, once stabilized, the patient underwent an internal cardioverter defibrillator (ICD) placement.

## Introduction

Marijuana is an illicit drug, which is increasingly abused in the United States due to state-specific variations in legalization and changing public opinion. Prevalence of usage increased from 4.1% in 2001-2002 to 9.5% in 2012-2013 [1]. Self-reported use is highest among adults between 18 to 25 years of age [2]. Marijuana exerts its effect on the body through the cannabinoid receptors CB1 and CB2. In addition to tetrahydrocannabinol (THC), marijuana has over 400 active chemicals, many of which have unknown effects on the body [3]. This raises critical concerns about the user's health as cardiac receptors may be stimulated or inhibited by these chemicals. The usual effects of marijuana on the cardiovascular system consist of dose-dependent tachycardia and hypotension, propagated primarily by THC [4]. In addition to this, various cardiac arrhythmias have been well documented, including atrial fibrillation and flutter, ventricular tachycardia and fibrillation, atrioventricular (AV) block, and asystole [5,6]. Current research on cardiac ion channel effects is an emerging area of study. Previous publications have shown an association between acute marijuana use and the presentation of Brugada syndrome [7]. The case presented here represents a classical type 1 Brugada pattern most likely caused by marijuana use.

## Case presentation

**Figure 1:**
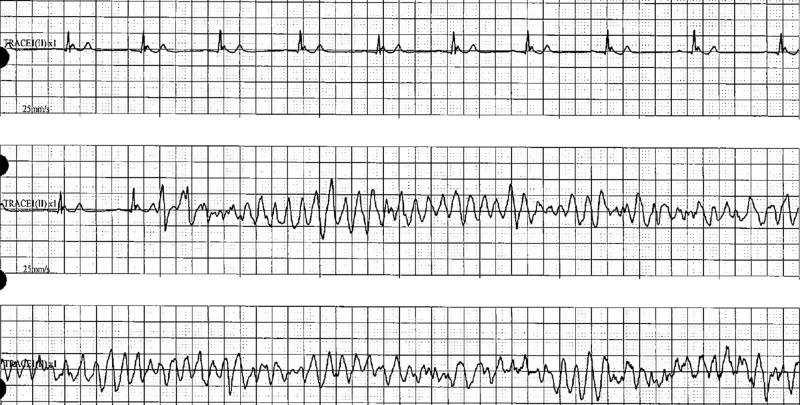
Sinus rhythm with spontaneous polymorphic ventricular tachyarrhythmia

**Figure 2:**
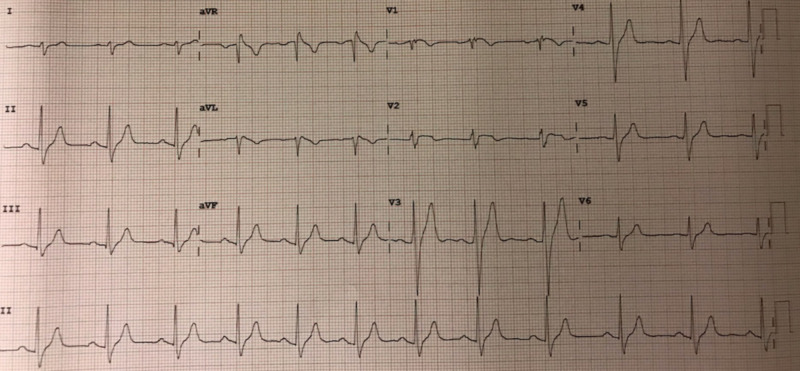
EKG with leads in normal position demonstrates a saddleback (type II) Brugada abnormality in V2. QT interval is within normal limits EKG: electrocardiogram

**Figure 3:**
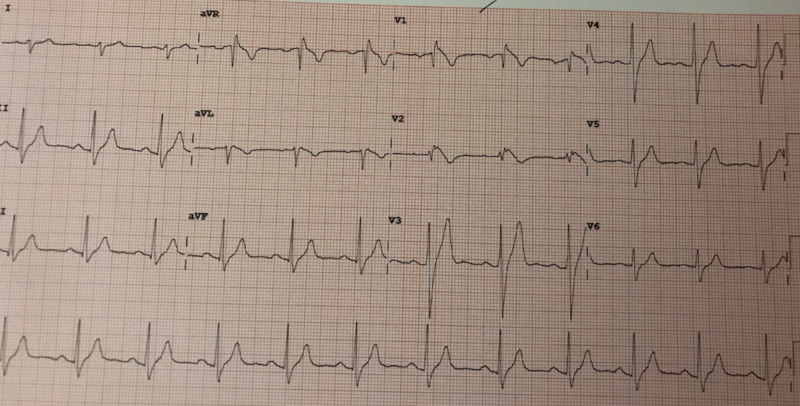
EKG with leads V1 and V2 placed in second intercostal space producing classic type I Brugada pattern EKG: electrocardiogram

A 22-year-old African-American male with a medical history of childhood asthma presented to the ED after suffering from a witnessed syncopal event. The patient had prior consumption of marijuana throughout the evening. He denied the use of any other medications. The presentation history was provided by the patient's girlfriend. She stated that she had witnessed the patient experience a syncopal event while on the couch. The patient recalled falling asleep on the couch and, upon waking, had found his girlfriend standing over him. There was no postictal confusion state and no report of seizure-like activity. Upon arriving at the ED, the patient went into a spontaneous, transient pattern of ventricular tachycardia and torsades de pointes with pulse (Figure 1). After these rhythms were aborted with amiodarone and magnesium, the patient converted into new-onset atrial fibrillation. Further inquiry into the patient’s family history revealed that two of his maternal aunts had internal cardioverter defibrillators (ICDs) implanted for unknown reasons. There was no other history of ICD, Brugada, or sudden cardiac death in the family. Notable workup in the ED revealed detectable but trivial serial troponins, no electrolyte abnormality, and urine drug screen (UDS) positive for cannabis. A 2D echocardiogram showed preserved left ventricular ejection fraction (LVEF) of 64% without structural and functional abnormality. An electrocardiogram (EKG) was obtained, which showed classic type II “saddleback” morphology in V2 (Figure 2). A second EKG was obtained with anterior leads V1 and V2 elevated approximately 2 cm on the chest, over the second intercostal space, resulting in classic Brugada type 1 pattern (Figure 3). The patient was monitored in the ICU, and care was taken to maintain potassium levels above 4 mmol/L and magnesium above 2 mmol/L, and to maintain core body temperature below 38 °C. The patient underwent ICD placement and was cautioned against further use of marijuana; he was also prescribed several other medications and advised on certain conditions as per The Brugada Foundation guidelines [8].

## Discussion

In a study by Patel SS et al., Brugada syndrome was found to have an incidence of 0.012-0.4% based on two samples among the urban population in the United States [9]. With the rise in popularity of marijuana, both for recreational use and medicinal purposes, its association with Brugada syndrome has raised questions about its safety profile. The primary chemical in marijuana, THC, exerts its effect primarily on CB1 and CB2 receptors, yet it is unclear if this is the direct trigger for Brugada morphology on EKG.

One trial from 2007 suggested that cannabinoid receptor antagonists offered protection against ischemia. In the study, myocardial injury in rats was induced by coronary artery occlusion followed by reperfusion. After this, the incidence of hypotension, ventricular arrhythmias, and the size of infarct were compared. Although the CB1 receptor antagonist did not show improvement from the control, activation of the CB2 receptor did show protective effects by reducing both infarct size and incidence of arrhythmia [9]. Additionally, an endogenous cannabinoid known as anandamide has been shown to inhibit the iNa+ current in neuronal cells but has shown no demonstrable effect on cardiac sodium channels [10,11]. The results from these trials indicate that a chemical substance found in marijuana, which is separate from THC, could be responsible for the perpetuation of Brugada pattern in primed individuals with abnormal SCN5A and SCN10A cardiac sodium channels. This is especially significant for our patient due to his race. A polymorphism in the SCN5A-S1103Y gene has increased frequency in African-Americans, which puts them at increased risk for sudden cardiac death [12].

A distinct correlation has been made between marijuana use and increased supraventricular and ventricular ectopic activity [13]. With the increasing abuse of this drug and the recent spread of its legalization, the potential for more cases of cannabinoid-induced Brugada syndrome is expected to rise concurrently. Currently, marijuana is described as a class IIb medication on BrugadaDrugs.org. As more cases like these emerge, it is important to re-evaluate the class status of marijuana to increase awareness about its potential side effects. This case highlights the importance of obtaining a very focused cardiac family history if a patient acknowledges the use of this substance. Additionally, patients who are actively smoking marijuana with a suspicious family history should be evaluated with EKG to screen for this deadly disease. This particular patient was extremely fortunate to have not suffered sudden cardiac death.

## Conclusions

In this report, we discussed a classic presentation of Brugada syndrome with a high potential for sudden cardiac death. The aim of this case presentation was to spread awareness about this condition, highlight the unintentional consequences of using seemingly harmless drugs (irrespective of whether they are illicit or recreational), and to call attention to one of the most rapidly evolving psychoactive drugs (which is still illicit in many US states and other countries) in current use.
